# Burnout Syndrome During COVID-19 Second Wave on ICU Caregivers

**DOI:** 10.2478/jccm-2022-0026

**Published:** 2022-11-12

**Authors:** Anaëlle Caillet, Marina Fillon, Margaux Plou, Emmanuel Tisson, Charles-Hervé Vacheron, Bernard Allaouchiche

**Affiliations:** 1 Hospices Civils de Lyon, Hospital center Lyon-Sud, ICU, F-69310, Pierre-Bénite, France; 2 Biostatistics-Bioinformatics Department, Public Health Unit, Hospices Civils de Lyon, Lyon, France; 3 CIRI, International Center for Research in Infectiology (Emerging Pathogens Laboratory Team), Inserm, U1111, Claude Bernard Lyon 1 University, CNRS, UMR5308, Lyon, France; 4 University Claude Bernard, Lyon1, Lyon, France; 5 Lyon university, VetAgro Sup, Lyon veterinary campus, UPSP 2016.A101, Pulmonary and Cardiovascular Agression in Sepsis, F-69280, Marcy l’Étoile, France

**Keywords:** psychological impact, burnout, COVID-19, ICU, caregivers

## Abstract

**Objective:**

The main objective of this article is to evaluate the prevalence of burnout syndrome (BOS) among the Intensive Care Unit (ICU) healthcare workers.

**Methods:**

The COVID-impact study is a study conducted in 6 French intensive care units. Five units admitting COVID patient and one that doesn’t admit COVID patients. The survey was conducted between October 20th and November 20th, 2020, during the second wave in France. A total of 208 professionals responded (90% response rate). The Maslach Burnout Inventory scale, the Hospital Anxiety and Depression Scale and the Impact of Event Revisited Scale were used to study the psychological impact of the COVID-19 Every intensive care unit worker.

**Results:**

The cohort includes 208 professionals, 52.4% are caregivers. Almost 20% of the respondents suffered from severe BOS. The professionals who are particularly affected by BOS are women, engaged people, nurses or reinforcement, not coming willingly to the intensive care unit and professionals with psychological disorders since COVID-19, those who are afraid of being infected, and people with anxiety, depression or post-traumatic stress disorder. Independent risk factors isolated were being engaged and being a reinforcement. Being a volunteer to come to work in ICU is protective. 19.7% of the team suffered from severe BOS during the COVID-19 pandemic in our ICU. The independent risk factors for BOS are: being engaged (OR = 3.61 (95% CI, 1.44; 9.09), don’t working in ICU when it’s not COVID-19 pandemic (reinforcement) (OR = 37.71 (95% CI, 3.13; 454.35), being a volunteer (OR = 0.10 (95% CI, 0.02; 0.46).

**Conclusion:**

Our study demonstrates the value of assessing burnout in health care teams. Prevention could be achieved by training personnel to form a health reserve in the event of a pandemic.

## Introduction

Currently, the world is experiencing an unprecedented health crisis. COVID-19 has spread to all continents in several successive waves and has affected almost all the world [1]. On may 2021, in France, we counted 5,667,324 confirmed cases, including 109,557 declared deaths [2]. The pandemic has deeply challenged our health systems organization, particularly among intensive care unit (ICU), which had to constantly readapt [3]. In France, at the peak of the pandemic, intensive care beds have strongly increased [4].

This massive flow of patients has imposed on hospitals an unprecedent reorganization. This caused an increased of ICU beds with a geographical, material and human adaptation in capacities. This need for human resources leaded underqualified healthcare worker in the ICU, and management adaptation in a context of sanitary emergency [5, 6]. In a previous study, these organizational changes required that mobilized people acquire specific ICU qualification in a short period of time. This led to an increase in anxiety, depression and burn out. Additionally, personal and family organizational disruptions for all concerned staff (day/night alternation, reorganization of schedules, family disruptions) [7].

Before the health crisis, ICU caregivers already were at risk of psychological disorders related to the difficulties of the emotional, organizational, and social situations encountered during the care. According to an American survey on post-traumatic stress disorder and burn out, among 332 nurses 16 % presented anxiety, 13% suffered from depression, 22% of nurses had symptoms of post-traumatic syndrome disorder and 18% met the diagnostic criteria for PTSD. A total of 86% of nurses had symptoms consistent with moderate burnout [8].

Thus, the COVID-19 exacerbated a pre-existing issue: according to the same study, from May to June 2020, 38% of ICU nurses were experiencing burnout. In Belgium,over a similar period, 68% of 1135 ICU nurses suffered from burnout [9].

Also, the literature shows a recurrence of certain risk factors of mental disorders identified: fear of being infected (lack of individual protective equipment), inability to rest, inability to care for family, experiencing difficult emotions, inadequate visiting policies, witnessing hasty decision-making, end-of-life settings [10]...

It is in this context of psychological tension for the staff that we decided to conduct the COVID Impact study.

The main objective of this article is to evaluate the prevalence of burnout syndrome (BOS) in the ICU team during the COVID-19 pandemic. Moreover, we estimated the risk factors associated with severe BOS.

## Materials and Methods

### Ethics Committee Agreement

This study has obtained the agreement of the ethics committee of Nimes (ref 20.0026). No written informed consent was required. The authors guarantee the anonymization of all data collected.

### Survey with data collection

The COVID Impact study was conducted during the second wave of COVID-19 in France. First, we developed a survey, composed of two parts: caregiver characteristics and scales. We used the Hospital Anxiety and Depression Scale (HADS) [11, 12, 13]. The surveys were distributed between October 20th, 2020 and November 20th, 2020. Six intensive care units participated: one which had no COVID patients and five admitting only COVID-19 patients. All staff working in the ICU had the survey delivred. A total of 208 professionals completed the survey.

### Scale used

The HADS is used to evaluate anxiety and depressive symptomatology and severity of symptoms. The scale is composed of 14 items, 7 for anxiety and 7 for depression. Every question has 4 responses, all from 0 to 3. The minimum possible score is 0, the higher score is 42. The scores for the anxiety questions and the depression questions are added together to obtain 2 scores that are then added together. [12]

– Absence of anxiety and depressive disorders: from 0 to 7
– Suspected anxiety or depressive disorders: from 8 to 10
– Proven anxiety or depressive disorders: from 11 to 21
– Existence of anxiety-depressive syndrome: from 15 to 42

The Impact of Event Revisited Scale (IES-R) was used to assess Post Traumatic Stress Disorder. The scale is composed of 22 items, each statement having a score between 0 and 4. then the scores of all the items must be added together. The minimum possible score is 0 and the higher is 88.

– Mild symptoms: from 0 to 32
– Moderate and severe symptoms: from 33 and more

Maslach Burnout Inventory (MBI) is a 22-item scale. The respondent answers questions related to his feelings and experiences at work over the last 7 days. This scale evaluates 3 components of burnout: the emotional exhaustion (EE), the depersonalization (DP) and the personal accomplishment (AP).

For the EE dimension, a score between 0 and 16 corresponds to a low level of EE. A score between 17 and 26 corresponds to a moderate level of EE and a score greater than or equal to 27 corresponds to a high level of EE. For the DP dimension, a score between 0 and 6 corresponds to a low level of DP. A score between 7 and 12 corresponds to a moderate level of PD and a score greater than or equal to 13 corresponds to a high DP. For the AP dimension, a score greater than or equal to 37 corresponds to a low level of AP. A score between 31 and 36 corresponds to a moderate level of AP and a score between 0 and 30 corresponds to high AP.

A high EE or DP score or a low AP score is sufficient to speak of burnout. Depending on the number of dimensions affected, there are stages of severity. The degree of burnout is as follow:

– Low: only 1 dimension is affected
– Moderate: ⅔ dimensions are affected
– Severe: 3 dimensions are pathological [14]

### Statistical analysis

Descriptive statistics were performed using median [interquartile range, IQR], and frequency (percentage) for qualitative variables. Differences between groups were estimated using the Wilcoxon Rank Sum Test for quantitative variables, and by the Chi-squared test for qualitative variables or Fisher test when applicable. In order to select the predictor of severe burn-out, a bidirectional stepwise regression analysis based on the Akaike information criterion was performed. For the modelling strategy, we removed the answer from the physician, because of convergence problems (None of the 25 Medical staff presented a severe burnout). From the final model, we estimated the odds ratio associated with their 95% confidence interval and respective p value, and to assess the calibration of the model, the c statistic from the model was estimated. Missing variables were imputed for the regression using multivariate imputation by chained equations. Statistical significance for the p value was set at 0.05. Statistical analysis was performed by using R software V 3.6.3 and the package survival.

## Results

A total of 208 professionals completed the survey; the team was composed of 230 professionals, for a response rate of 90%.

### Cohort Description and univariate analysis

The cohort (Table 1) includes 208 professionals, 152 women (73.4%), the most represented age group is under 30 years old with 99 professionals (47.8%). One hundred and twenty-six people were married (61.5%). Among the cohort, 52.4% (109 people) were caregivers and 12% (25 people) were physicians. Forty-nine percent of respondents did not work in the ICU before the COVID-19 pandemia. During the first wave, 69.7% (145 people) were already in ICU, 30.3% (63 people) weren’t volunteer to work in ICU, and 58.7% had less than 12 months of critical care experience. During the first wave in April, 65.2% (162 people) of the cohort was in COVID+ ICU compared to 86.9% (179 people) in November. A total of 29.9% (56 professionals) did not feel trained enough. Approximately 10% (23 people) of the cohort had started psychological follow-up since the beginning of COVID-19. One hundred and two professionals (55.7%) felt that institutional psychological support was insufficient. Nineteen-point seven percent (41 people) of the team had severe BOS.

**Table 1:** Univariate analysis and cohort description according burnout

Characteristics		Low and moderate BOS N = 167	Severe BOS N = 41	p-value	Total N = 208
Sex					
	Women	116 (69.9)	36 (87.8)	0.033	152 (73.4)
	Men	50 (30.1)	5 (12.2)		55 (26.6)

Marital status					
	Single	71 (43.3)	8 (19.5)	0.009	79 (38.5)
	Engaged	93 (56.7)	33 (80.5)		126 (61.5)

Job					
	Other	63 (37.7)	12 (29.3)		75 (36.1)
	Reinforcement	9 (5.4)	7 (17.1)	0.012	16 (7.7)
	Nurse	71 (42.5)	21 (52.1)		92 (44.2)
	Doctors	24 (14.4)	1 (2.4)		25 (12)

Initial Department					
	Other	22 (13.3)	2 (4.9)		24 (11.7)
	Operatory room	34 (20.6)	10 (24.4)		44 (21.4)
	Trauma room	4 (2.4)	0 (0)	0.028	4 (2)
	Intensive Care Unit	84 (50.9)	23 (56.1)		107 (51.9)
	Recovery room	1 (0.6)	4 (9.8)		5 (2.4)
	Intermediate care unit	20 (12.2)	2 (4.9)		22 (10.7)

Volunteer					
	Yes	63 (38.7)	3 (7.3)	<0.001	66 (32.4)
	Not concerned	73 (44.8)	22 (53.7)		95 (46.6)

Medical history of psychological disorders					
	Yes, before COVID-19	13 (7.8)	2 (4.9)	0.029	15 (7.2)
	Yes, after COVID-19	2 (1.2)	4 (9.8)		6 (2.9)

Fear of being infected					
	Yes	71 (43.3)	26 (63.4)	0.033	97 (47.3)

Management of dead patient during 1st wave					
	Yes	84 (77.8)	24 (22.2)	0.44	108 (51.9)

Thinks he might needs psychological help				<0.001	
	Yes	10 (6.1)	17 (42.5)		27 (13.2)

Thinks that institutional psychological supports are sufficient				0.113	
	Yes	69 (85.2)	12 (14.8)		81 (44.3)

Consult a psychologist in town					
	Yes	4 (2.4)	6 (14.6)	0.005	10 (4.8)

Describe ICU experience as a trauma				<0.001	
	Yes	9 (20.9)	8 (88.9)		17 (32.7)

Results are expressed as a percentage. BOS: Burnout syndrome

Table 1 presents univariate analysis results. Women were more affected by BOS 24% VS 9% (p=0.033), engaged professionals were more affected by BOS 26% VS 10% (p=0.009). Only 4% of doctors have been affected by BOS VS 44% of the reinforcement team and 23% of nurses (p=0.012). When professionals didn’t come to the ICU voluntarily, they were more affected by BOS 37% VS 5% (p<0.001). The fear of being contaminated increases the risk of developing burnout 27% VS 14% (p=0.033). When the professional thinks he or she might need psychological help, burnout prevalence increases (p<0.001). It’s the same if the professional describes his experience in ICU as a trauma for himself (p<0.001).

**Fig. 1:**
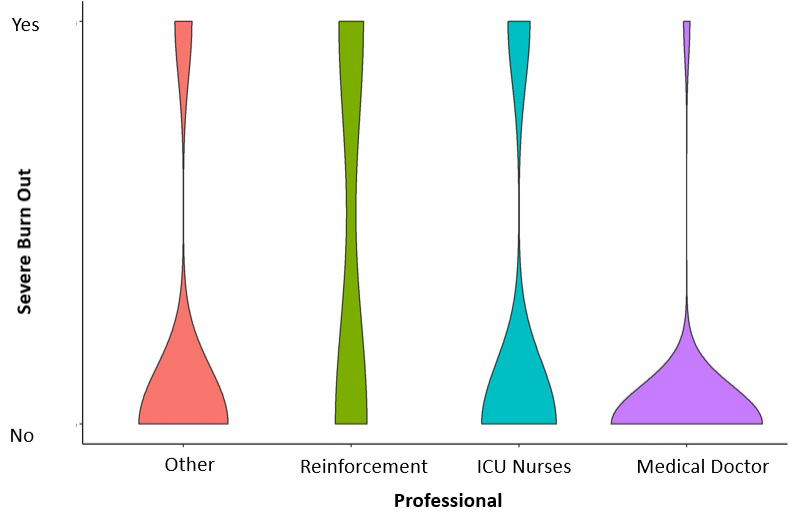
Distribution of severe bornout syndrom according to job occupation

Figure 1 shows the distribution of severe BOS according to the professional job.

Concerning anxiety: 48% of the professionals who suffer from an anxiety disorder also suffer from severe burnout. When the professionals were not anxious, only 6% suffered from severe BOS (p<0.001).

Concerning the anxio-depressive syndrome, 56% of the professionals who suffer from an anxio-depressive syndrome suffer from severe burnout. When the professionals did not suffer from this syndrome, only 7% suffered from a severe BOS (p<0.001).

Concerning PTSD, 53% of the professionals who suffer from PTSD suffer from severe burnout. When professionals do not suffer from PTSD, only 11% suffer from severe BOS (p<0.001).

### Multivariate analysis

Important to notice: there was no BOS for doctors, they’ve been excluded from multivariate analysis.

We observed a high correlation between EE and PTSD, anxiety, and Depressive symptomatology, and a moderate correlation between DP and AP and the PTSD, anxiety, Depressive symptomatology (Table 3) The independent risk factors for BOS are (Table 2).

**Table 2:** Multivariate analysis

Variable	Units	Odds Ratio		CI.95	p-value
Marital status	Single	Ref			
	Engaged		3.61	[1.44 ;9.09]	0.006303

Job	Doctors	Ref			
	Other		6.13	[0.70;53.90]	0.102214
	Reinforcement		37.71	[3.13;454.35]	0.004254
	Nurses		7.09	[0.85;59.30]	0.070504

Management of dead patient during 1st wave	No	Ref			
	Yes		1.94	[0.88;4.30]	0.100296

Thinks that institutional psychological supports are sufficient	Yes	Ref			
	No		1.87	[0.84;4.17]	0.123360

Volunteer	Not concerned	Ref			
	No		1.02	[0.41;2.50]	0.970734
	Yes		0.10	[0.02;0.46]	0.002885

**Table 3:** Correlation table between Anxiety, Depression, Post Traumatic Stress Disorder and Emotional Exhaustion (EE), Depersonalization (DP), and the personal accomplishment (AP).

		PTSD	Anxiety	Depressive symptomatology
EE	0.67	[0.59;0.74]	0.69 [0.61;0.76]	0.67 [0.59;0.74]
DP		0.47 [0.35;0.57]	0.46 [0.35;0.56]	0.37 [0.24;0.48]
AP		-0.25 [-0.37;-0.11]	-0.24 [-0.37;-0.11]	-0.41 [-0.51;-0.28]

Evaluation of the Post Traumatic Stress Disorder (PTSD) by IES-R scale, Anxiety and Depressive symptomatology by HADS scale. EE: emotional exhaustion; DP: depersonalization; AP : Personal Accomplishment, evaluated by the Maslach Burnout Inventory (MBI).

Being engaged (OR = 3.61 (95% CI, 1.44;9.09)

Don’t working in ICU when it’s not COVID-19 pandemic (reinforcement) (OR = 37.71 (95% CI, 3.13;454.35)

Being a volunteer (OR = 0.10 (95% CI, 0.02;0.46). This variable corresponds to 3 statuses: I am ICU personnel (a), I am a back-up and I do not come to help voluntarily (b), I am a back-up and I come to help voluntarily (c). Therefore, taking as a reference the ICU staff (a), I have no excess risk of being a non-volunteer (b) compared to the ICU staff, but it is a real protective factor to be a volunteer (c).

And finally, it is important to point out that paramedics have a real excess risk that is not highlighted here due to lack of power.

## Discussion

The cohort of the COVID Impact study includes 208 professionals, 52.4% are caregivers. 19.7% of the respondents suffered from severe BOS. The risk factors for BOS are: being engaged, being a nurse, don’t working in ICU when it’s not COVID-19 pandemic, having managed patients who died during the first wave, thinking that institutional psychological care supports are insufficient and do not being volunteer.

The COVID Impact study, contrary to the other studies, makes a distinction between the levels of BOS. To give the results, we chose to divide the sample in two: low/moderate BOS and severe BOS. This distinction is not often made in the literature.

Many studies have been conducted on BOS before Coronavirus health crisis. Between 25% and 46% of physicians and between 70% and 80% of nurses are suffering from BOS [8, 15, 16, 17, 18]. Nearly 50% of the health care workers suffered from BOS [22, 23]. Indeed, the prevalence of BOS fluctuates depending on the geographic area, healthcare system and culture [3]. The most identified risk factors in the literature were being female, being a nurse, working on the front line with exposure to COVID-19, lack of experience, comorbidities, night shifts, nurse/patient ratio, length of work week, and shortage of staff and equipment intending to leave work, being young, being a nurse, and having low life satisfaction [3, 9, 21, 23] , [15, 19]. Many organizational factors such as workload, turnover, weekly work time, nurse-patient ratio, level of training, internal team conflicts and conflicts with patients are also involved in the development of psychological distress [15, 19, 20]. Another psychological comorbidity increases the risk of BOS [15, 17]. The risk of developing a psychological disorder in the ICU is 32.3%. Therefore, the risk of developing a BOS is increased when the caregivers are working in the ICU [19]. Even if the prevalence of burnout varies in the literature, this requires both institutional and managerial reaction during these stressful periods. Notably, we need to routinely offer psychological support to professionals.

To prevent BOS, several solutions are currently explored. It is necessary to identify profiles at-risk by using all the risk factors identified in the literature [7, 9, 25]. Then, it is necessary to propose to the whole team psychological care centered on the management of emotions, stress, reflective practices and relaxation exercises [16, 17, 19, 24, 25, 26]. The optimization of communication is also essential with debriefings in order to encourage exchanges with the physicians and to optimize communication with the management team [16, 17, 24, 26]. Facilitating collaboration between teams and human resource managers will facilitate the prevention and management of psychological disorders such as BOS [16]. Increased training could also decrease the prevalence of BOS [17]. The last point of solution is the development of resilience. Indeed, this ability would decrease in a statistically significant way disorders such as PTSD, anxiety, depression and BOS. Individual factors that facilitate resilience are having a social life, being positive, being able to communicate, being emotionally balanced, not having conflicts at work, being trained, having the patient’s wellbeing as a common goal, and having material resources [18, 27].

The main strengths of the study are that it is conducted in both COVID and non-COVID units and the heterogeneity of the cohort. The main limitation is that the study has been conducted in 6 different ICU but in only one hospital center.

## Conclusion

The COVID Impact study shows that 80% of healthcare professionals suffer from low or moderate BOS and 20% suffer from severe BOS. Regarding the nurses, the prevalence of BOS is around 70% [9] in the literature, the study conducted in Lyon found 50% of severe BOS among nurses.

The risk factors found in the Covid Impact study are similar: being a woman, being engaged, lack of experience and assignment outside of ICU before health crisis, psychological disorders and the risk of contamination.

Prevention could be achieved by training personnel to form a health reserve in the event of a pandemic.
